# Long-term sedation with remimazolam besylate versus propofol in critically ill patients during invasive mechanical ventilation: a study protocol for a multicenter randomized non-inferior trial

**DOI:** 10.3389/fphar.2023.1139872

**Published:** 2023-07-28

**Authors:** Xiaobo Yang, Yun Tang, Ruofei Du, Yuan Yu, Jiqian Xu, Jiancheng Zhang, Hong Liu, Xiaojing Zou, Lehao Ren, Shiying Yuan, You Shang

**Affiliations:** ^1^ Department of Critical Care Medicine, Union Hospital, Tongji Medical College, Huazhong University of Science and Technology, Wuhan, Hubei, China; ^2^ Department of Biostatistics, University of Arkansa for Medical Sciences, Little Rock, AR, United States

**Keywords:** remimazolam, propofol, sedation, intensive care, mechanical ventilation

## Abstract

**Background:** Remimazolam besylate is a novel ultra-short-acting benzodiazepine that can potentially be a safe and effective sedative in intensive care units. This study aims to assess whether remimazolam besylate is not inferior to propofol in maintaining mild-to-moderate sedation in critically ill patients receiving long-term mechanical ventilation.

**Methods and analysis:** This is a multicenter, randomized, single-blind, propofol-controlled, non-inferiority study. Eligible patients are randomly assigned to receive remimazolam besylate or propofol in a 1:1 ratio to maintain a Richmond Agitation–Sedation Scale score between −3 and 0. When patients are under-sedated, rescue sedation of dexmedetomidine is added. The primary outcome is the percentage of time in the target sedation range. The secondary outcomes are hours free from the invasive ventilator in 7 days, successful extubation in 7 days, and weaning time, the length of intensive care unit stay, the length of hospital stay, and mortality in 28 days. Modified intention-to-treat and safety analysis is performed.

**Clinical trial registration number:** https://clinicaltrials.gov/ct2/show/NCT05555667.

## Background

Analgesia and sedation are important treatments in intensive care units (ICUs), which relieve pain and physical discomfort, reduce adverse stimulation of mechanical ventilation, and prevent agitation-related harm (Devlin et al., 2018). Midazolam and propofol are two of the most commonly used sedatives in current clinical practice. Unwanted hypotension in already hemodynamically unstable critically ill patients mainly restricts their wide use in the ICU (Ostermann et al., 2000). Another concern is propofol-related infusion syndrome, a lethal condition associated with prolonged administration of propofol and characterized by multiple organ system failures (Roberts et al., 2009). The potential predisposing risk factors include increased levels of catecholamine, the concomitant use of steroids, and increased serum lipids, which are common in critically ill patients receiving long-term mechanical ventilation. Various manifestations of propofol-related infusion syndrome also coincide with the pathophysiological disturbances of these patients, making it hard to differentiate. Outbreaks of postoperative infection traced back to contaminated propofol were reported in 1995 (Bennett et al., 1995). Infections caused by the contamination of propofol are a complex issue of public health and public interest, which have been reported in industrialized countries more than in developing countries. Under-recognition and lack of surveillance are responsible for the discrepancy between reality and medical literature (Zorrilla-Vaca et al., 2016). Compared with propofol, midazolam is associated with fewer cardiorespiratory depression events but a longer time of recovery and mechanical ventilation after cessation of continuous infusion (Devlin et al., 2018; Garcia et al., 2021).

Remimazolam besylate is a novel, ultra-short-acting benzodiazepine approved for the induction and maintenance of general anesthesia in Japan and South Korea and for procedural sedation in the United States, China, and Europe (Goudra and Mason, 2021). Remimazolam besylate undergoes organ-independent metabolism and is hydrolyzed by tissue esterases into an inactive metabolite, making it a potential sedative that can be used in patients with impaired liver or renal function (Stöhr et al., 2021; Antonik et al., 2012). Long-term infusion and high doses are unlikely to cause accumulation or extended effects (Lohmer et al., 2020). It can also be antagonized by flumazenil. Recently, there has been a growing interest in exploring the use of remimazolam in ICUs (Song et al., 2022; Liu et al., 2021).

Our phase I study was a dose-finding study of 36 mechanically ventilated non-cardiac surgical patients, which showed that a continuous infusion of remimazolam besylate between 0.125 mg/kg/h and 0.15 mg/kg/h for 8–24 h provided a light-to-moderate level of sedation with good efficacy, a rapid onset, and an excellent safety profile (Tang et al., 2022a). Our pilot study, including 30 patients requiring prolonged mechanical ventilation, revealed that it was feasible to use remimazolam besylate to maintain light-to-moderate sedation and that compared with propofol, no differences were identified in terms of the proportion of time in the target sedation range without using a second sedative, ventilator-free days at day 7, the length of ICU stay, 28-day mortality, or adverse events, such as bradycardia and hypotension (Tang et al., 2022b).

In the present study, based on the results of our previous studies, we aim to assess whether remimazolam besylate is non-inferior to propofol in maintaining mild-to-moderate sedation in critically ill patients receiving long-term mechanical ventilation.

## Registration

This study was registered at ClinicalTrials.gov (NCT05555667) on 26 September 2022 (https://clinicaltrials.gov/ct2/show/NCT05555667).

## Materials and methods

### Study design

**FIGURE 1:**
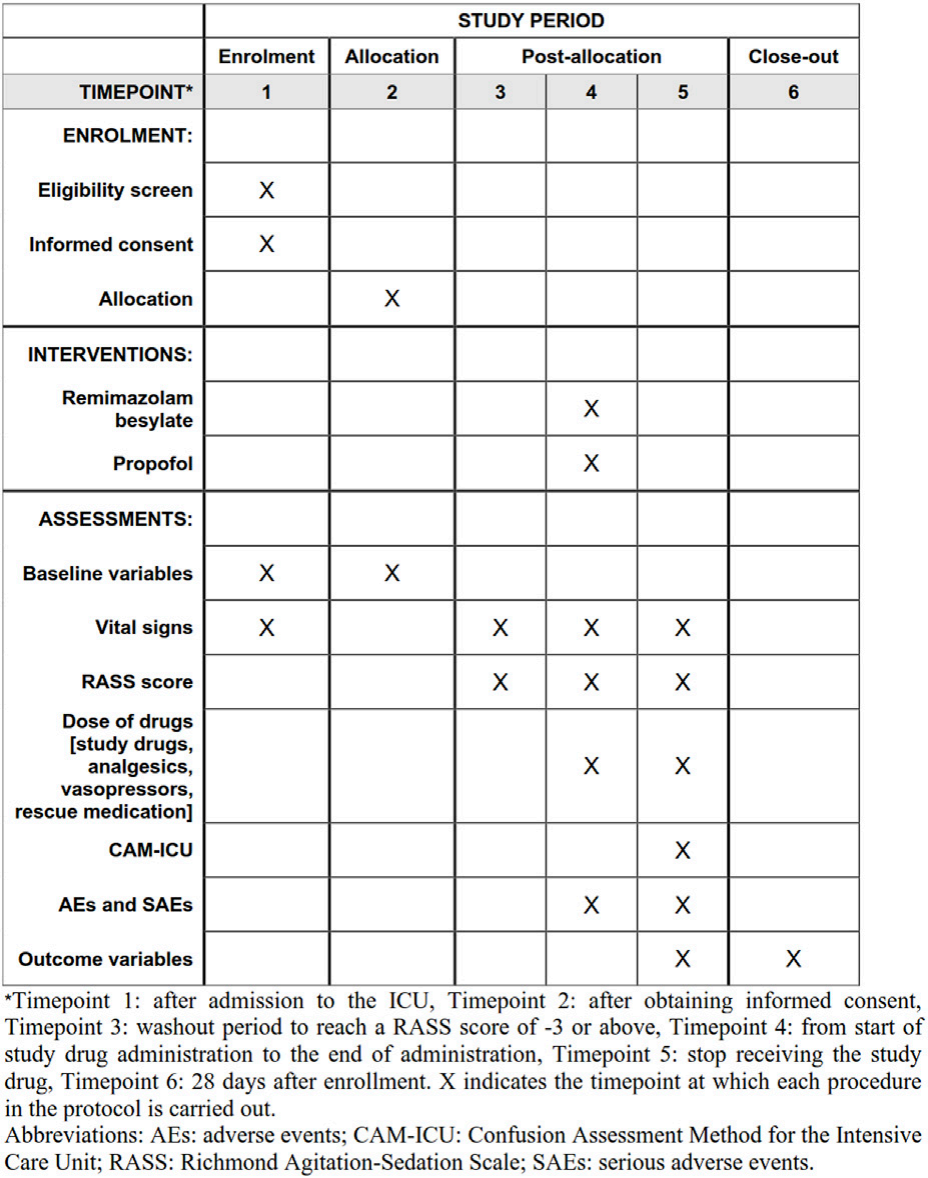
Standard Protocol Items: Recommendations for Interventional Trials (SPIRIT) schedule for enrollment, interventions, and assessments.

This is a multicenter, randomized, single-blind, propofol-controlled, non-inferiority study initiated by Wuhan Union Hospital. Apart from our ICU, 11 ICUs from tertiary hospitals in China enrolled patients in the trial. The schedule of enrollment, interventions, and assessments is reported according to the Standard Protocol Items: Recommendations for Interventional Trials (SPIRIT) statement (Figure 1).

### Inclusion criteria

Inclusion criteria are as follows: 1) age ≥18 years and ≤80 years, 2) intubated within 96 h before enrollment and expected to require mechanical ventilation and sedation for at least 24 h, 3) a need for light-to-moderate sedation defined as a target sedation Richmond Agitation–Sedation Scale (RASS) score between 0, alert and calm, and −3, responds to verbal stimulation by movement or eye opening to voice but no eye contact (Sessler et al., 2002), and 4) written informed consent is obtained from the patient or a legal representative.

### Exclusion criteria

Exclusion criteria are as follows: 1) body mass index (BMI) < 18 or >30 kg/m^2^, 2) acute severe neurological disorder and any other condition complicating RASS assessment, 3) systolic blood pressure less than 90 mm Hg after appropriate intravenous volume replacement and continuous infusions of two kinds of vasopressors, 4) heart rate less than 50 beats/min or second- or third-degree heart block in the absence of a pacemaker, 5) unstable angina or acute myocardial infarction, 6) left ventricular ejection fraction less than 30%, 7) contraindicate or allergic to study drugs, 8) expected death within 48 h of enrollment or lack of commitment to aggressive treatment by family or the medical team, 9) scheduled for surgery within 24 h, 10) acute hepatitis or serious hepatic dysfunction (Child–Pugh class C), 11) chronic kidney disease with glomerular filtration rate (GFR) < 30 mL/min/1.73 m^2^, 12) alcohol abuse, 13) myasthenia gravis, 14) pregnancy or lactation, and 15) any other condition considered unsuitable for this trial by treating physicians or researchers.

### Randomization and blinding

Eligible patients of both genders are randomly assigned to the remimazolam group or the propofol group in a 1:1 allocation ratio using random sequences of block sizes of 2, 4, 6, 8, or 10 stratified by the participating center. No dummies are used, so treating physicians and researchers are not blinded because of the different appearances of the two drugs. Patients are effectively blinded because drugs are given when the patients are sedated.

### Intervention

Analgesics and sedatives used before enrollment are discontinued. Patients receive a continuous intravenous infusion of remifentanil at 4.0 μg/kg/h for analgesia prior to the study drug administration. The study drug administration is initiated when patients have a baseline RASS score of −3 or above for the first time after enrollment.

Patients in the remimazolam group receive remimazolam besylate (Yichang Humanwell Pharmaceutical Co., Ltd., China) intravenously at an initial rate of 0.2 mg/kg/h, which is titrated up (maximum of 1.0 mg/kg/h) or down at 0.05 mg/kg to achieve the target sedation level. Patients in the propofol group receive propofol (Fresenius Kabi China Co., Ltd.) intravenously at an initial rate of 2.0 mg/kg/h, which is titrated up (maximum of 4.0 mg/kg/h) or down at 0.5 mg/kg to achieve the target sedation level. If the RASS score > 0 and the rate of remimazolam besylate ≥ 0.5 mg/kg/h or propofol ≥ 3 mg/kg/h, the rate of remifentanil is titrated up at 1.5 μg/kg/h every time concomitantly with the titration up of remimazolam besylate or propofol. The maximum rate of remifentanil is 9.0 μg/kg/h. If the RASS score < −3, the rate of remifentanil is titrated down at 1.5 μg/kg/h every time concomitantly with the titration down of remimazolam besylate or propofol.

Assessment of the RASS score is performed every 4 h. Boluses (remimazolam besylate 0.05–0.1 mg/kg or propofol 0.3–0.5 mg/kg in 30 ± 10 s) or temporarily stopping the infusion of the study drugs is at the discretion of treating physicians. If the maximum dose of the study drug is insufficient to sedate, rescue sedation of dexmedetomidine at 0.2–0.7 μg/kg/h is given intravenously.

Patients stop receiving the study drug if 7 days pass after enrollment, or when patients decease, require deep sedation, are scheduled for surgery, are discontinued from the study drug for at least 12 h by treating physicians, are discharged from the ICU, or are withdrawn from the trial, whichever comes first. Patients are followed up at 28 days.

### Outcomes

The primary outcome is the percentage of time in the target sedation range without rescue sedation of the total duration of the study drug infused.

The secondary outcomes include the following: 1) duration (in hours) free from invasive ventilator in 7 days, 2) successful extubation in 7 days, defined as extubation of the endotracheal tube for the first time in 7 days without reintubation or switching to tracheostomy within the following 48 h, 3) weaning time in minutes, defined as the interval between continuous discontinuation of the study drug and extubation in patients with successful extubation, 4) the length of stay in the ICU in 28 days, 5) the length of hospital stay in 28 days, and 6) mortality in 28 days.

All of the following that occurred before discontinuation of the study drug are considered as safety outcomes: 1) hypotension (systolic blood pressure less than 80 mmHg or diastolic blood pressure less than 50 mmHg for 5 min or treated with vasopressors), 2) hypertension (systolic blood pressure greater than 160 mm Hg or diastolic blood pressure greater than 100 mmHg for 5 min or treated with vasodilators), 3) bradycardia (heart rate less than 50 bpm for 5 min or treated with medication to increase heart rate), 4) tachycardia (heart rate greater than 120 bpm for 5 min or treated with medication to decrease heart rate), 5) unplanned extubation of the endotracheal tube, 6) shock, 7) hospital-acquired pneumonia, 8) myocardial infarction, 9) cerebral ischemic stroke, 10) cerebral hemorrhagic stroke, 11) pulmonary embolism, 12) upper digestive tract ulcer confirmed endoscopically, 13) more than 500 mL of bloody stool, 14) delirium assessed using the confusion assessment method for the intensive care unit (CAM-ICU) (Ely et al., 2001), 15) delta sequential organ failure assessment (SOFA) score, defined as the difference between the SOFA score when each patient stops receiving the study drug and the SOFA score at inclusion, and 16) withdraw from the trial because of intolerance, severe adverse events, or other safety concerns. All safety outcomes are monitored by the Data Safety Monitoring Committee (DSMC) consisting of two intensivists and one statistician.

### Data collection and management

Upon enrollment, demographics, clinical data (vital signs, pertinent laboratory, and arterial blood gas analysis), the severity of illness (APACHE II score and SOFA score), and comorbidity are collected. During sedation, all vital parameters, including blood pressure, heart rate, and peripheral oxygen saturation (SpO_2_), are monitored continuously. The RASS score, vital signs, and vasopressors are recorded every 4 h. An investigator and a bedside nurse assess the RASS score at the same time, and disagreements are resolved by consultation with a third medical staff. Duration (in hours) free from the invasive ventilator, cumulative doses of sedatives, analgesics, and use of rescue medications are recorded daily. Adverse effects and extubation in 7 days and ICU discharge, hospital discharge, and mortality in 28 days are registered.

A trained investigator at each center is responsible for daily patient screening, enrolling, ensuring adherence to the protocol, and completing the electronic case report form (e-CRF). Data are handled confidentially and anonymously; only authorized personnel are permitted to access data. Data accuracy is periodically monitored and verified by the principal investigator.

### Safety evaluation

Safety assessments are composed of monitoring vital signs during the study and observing and recording all adverse events (AEs) and serious adverse events (SAEs). AEs are defined as all unfavorable/unexpected medical events that occur in patients, whether causally related to the study drugs or not, for example, hypotension, hypertension, bradycardia, tachycardia, and unplanned extubation of the endotracheal tube from safety outcomes. SAEs are defined as any medical occurrence resulting in any of the following outcomes: a life-threatening condition, death, threat of causing permanent or significant disability or incapacity, or a condition that requires or prolongs patient hospitalization, for example, shock, hospital-acquired pneumonia, myocardial infarction, cerebral ischemic stroke, cerebral hemorrhagic stroke, pulmonary embolism, upper digestive tract ulcer confirmed endoscopically, and more than 500 mL of bloody stool from safety outcomes. All AEs are treated immediately. SAEs are reported to the local medical department and ethics committee within 24 h. In addition, researchers purchase clinical trial insurance, which compensates for treating any harm that occurs during this study.

### Estimation of sample size

Our pilot study demonstrated about 70% of the time in the target range of sedation without using rescue medications in remimazolam besylate and propofol groups (Tang et al., 2022b). The distribution of the percentage of time in the target range of sedation without using rescue medications was skewed, with 100% as the maximum value. A non-inferior two-staged group-sequential design with an equal number of patients in both groups is used, assuming a non-inferiority margin = 10%, α = 0.025, and power = 0.80. Three hundred and twenty patients in each group are needed when sample size estimation is conducted using the gscounts package of R (Mütze et al., 2019).The R package gscounts is available for download on the Comprehensive R Archive Network (CRAN). Considering 12% of patients in our pilot study with less than six RASS evaluations, an adjusted total number of 364 patients are needed in each group. A blinded interim analysis of the primary outcome is conducted by the statistician from the DSMC after 182 patients in each group are included. If superiority is non-significant at interim analysis, the trial continues to the next half.

### Statistical analysis

A modified intention-to-treat analysis of patients with at least six RASS evaluations is conducted. Continuous data are presented as means with standard deviations or medians with interquartile ranges (IQRs), and categorical data are presented as frequencies and proportions. A two-sided *p* < 0.05 is considered statistically significant. In addition to the treatment drugs, the count of RASS evaluation is a crucial covariable, and negative binominal regression is used to evaluate the primary outcome. For secondary outcomes and safety outcomes, continuous variables are analyzed using Student’s t-test or the Mann–Whitney U test based on the distribution, and categorical variables are analyzed by the Fisher exact test. The mortality of the two groups between inclusion and 28 days is presented using the Kaplan–Meier method, and a survival time analysis is conducted using the log-rank test. A sensitivity analysis of the primary outcome is conducted using Student’s t-test or the Mann–Whitney U test based on the distribution.

### Ethics approval and informed consent

The study protocol was approved by the Ethics Committee of Union Hospital (2022-0391-01) and will be followed by all participating centers. Procedures will be performed following the Declaration of Helsinki. Written informed consent will be obtained from all patients or their legal representatives before enrollment. Participation is voluntary, and patients’ privacy will be protected. Patients or their legal representatives have the right to withdraw from the study at any time for any reasons, and their care will not be affected.

### Dissemination policy

Research data can be presented or publicized in agreement with the principal investigator only. Study results will be published in a peer-reviewed journal and be presented to patients, clinicians, and the public during local, national, and international meetings and conferences.

## Discussion

The current study is a large-sample-sized, randomized, multicenter trial to evaluate the efficacy and safety of the use of remimazolam besylate in critically ill patients receiving long-term mechanical ventilation. If a non-inferior effect of mild-to-moderate sedation to propofol is shown, it will have a considerable impact on the future sedation of mechanically ventilated critically ill patients. Remimazolam besylate is a potential replacement for propofol and midazolam with a smaller chance of hemodynamical depression and contamination than propofol, no chance of propofol-related infusion syndrome, and a faster recovery than midazolam after cessation of continuous infusion.

Remimazolam besylate has a fast and short-acting characteristic similar to propofol and is expected to be a similarly effective sedative in the ICU with fewer metabolic concerns and cardiovascular depression events. Studies have shown that its effect on hemodynamics is more stable than that of propofol and can be safely used in patients with unstable circulation (Qiu et al., 2022; Tang et al., 2022c). Remimazolam is currently being tested in a clinical trial for sedation as an alternative to midazolam in ICU patients (Liu, 2021).

The dosages of remifentanil and propofol used in our trial are commonly recommended (Des et al., 2005; Ely et al., 2001). In critically ill patients, a low dosage of remifentanil (0.5/kg/min) leads to calmness, and higher dosages can be used for patients on ventilators (Franco et al., 2002). The dosage of remimazolam was chosen based on our studies (Tang et al., 2022a; Tang et al., 2022b).

This study has some limitations. First, it is difficult to conduct a double-blind trial because of the milk-like appearance of propofol. Second, the RASS score is subjectively evaluated, although the guidelines suggested that the RASS and SAS are the most valid and reliable sedation assessment tools for measuring the quality and depth of sedation in adult ICU patients (Devlin et al., 2018). To validate the sedation score assessment, an agreement on the RASS between two medical personnel is needed. Third, all participants are screened and enrolled during sedation, and pretrial sedation causes potential bias. Fourth, patients needing deep sedation are excluded, but these patients will be studied further (Yang, 2022). Fifth, as far as we know, phase II trials on remimazolam in patients with hepatic or renal impairment are scarce, and the ethics committee recommended against including these patients in this phase III trial.

## Data Availability

The original contributions presented in the study are included in the article/Supplementary Material; further inquiries can be directed to the corresponding author.
